# Realtime nowcasting with a Bayesian mixed frequency model with stochastic volatility

**DOI:** 10.1111/rssa.12092

**Published:** 2015-01-27

**Authors:** Andrea Carriero, Todd E. Clark, Massimiliano Marcellino

**Affiliations:** ^1^ Queen MaryUniversity of LondonUK; ^2^ Federal Reserve Bank of ClevelandUSA; ^3^ Bocconi UniversityMilanand Innocenzo Gasparini Institute for Economic Research Milan Italy; ^4^ Centre for Economic Policy ResearchLondonUK

**Keywords:** Bayesian methods, Forecasting, Mixed frequency models, Prediction

## Abstract

The paper develops a method for producing current quarter forecasts of gross domestic product growth with a (possibly large) range of available within‐the‐quarter monthly observations of economic indicators, such as employment and industrial production, and financial indicators, such as stock prices and interest rates. In light of existing evidence of time variation in the variances of shocks to gross domestic product, we consider versions of the model with both constant variances and stochastic volatility. We use Bayesian methods to estimate the model, to facilitate providing shrinkage on the (possibly large) set of model parameters and conveniently generate predictive densities. We provide results on the accuracy of nowcasts of realtime gross domestic product growth in the USA from 1985 through 2011. In terms of point forecasts, our proposal improves significantly on auto‐regressive models and performs comparably with survey forecasts. In addition, it provides reliable density forecasts, for which the stochastic volatility specification is quite useful.

## 1 Introduction

Nowcasting has come to be commonly viewed as an important and unique forecasting problem; see, for example, Banbura *et al*. (2011, 2013). It is important because current quarter forecasts of gross domestic product (GDP) growth and inflation provide useful summaries of recent news on the economy and because these forecasts are commonly used as inputs to forecasting models, such as some of the dynamic stochastic general equilibrium models in use at central banks, that are effective in medium‐term forecasting but not necessarily short‐term forecasting. As studies such as Faust and Wright (2009, 2013) have emphasized, initial quarter forecasts often play a key role in the accuracy of forecasts at subsequent horizons. Nowcasting is unique in that, to some degree, it involves ‘simply’ adding up information in data releases for the current quarter. A key challenge is dealing with the differences in data release dates that cause the available information set to differ over points in time within the quarter—what Wallis (1986) referred to as the ‘ragged edge’ of data.

The nowcasting method that we propose in this paper is motivated in part by three key findings in the broader forecasting literature. First, prior work, particularly De Mol *et al*. (2008), Banbura *et al*. (2010) and Carriero *et al*. (2011), has shown that, with large data sets, estimation with Bayesian shrinkage is a viable alternative to factor model methods. Second, Clark (2011), Carriero *et al*. (2012) and D’Agostino *et al*. (2013) found it useful for forecasting purposes to incorporate stochastic volatility in vector auto‐regressive (VAR) models, for both point and density forecasts. Third, some other prior work has shown that direct multistep methods of forecasting can be at least as accurate as iterated methods (e.g. Marcellino *et al*. (2006)) for multistep forecasting. At a forecast horizon of *h*>1, the direct approach rests on estimates of a model relating $y_{t+h}$ to information in period *t*. The iterated approach involves a model relating $y_{t+1}$ to information in period *t* and iterating forwards to obtain two‐step forecasts from one‐step forecasts, etc. The direct approach can be more accurate than the iterated approach in the presence of model misspecification and does not require modelling the behaviour of the explanatory variables, thus making univariate modelling sufficient.

Building on this past work, we develop a new Bayesian mixed frequency with stochastic volatility (BMFSV) model for point and density nowcasting. In particular, we produce current quarter forecasts of GDP growth with a (possibly large) range of available within‐the‐quarter monthly observations of economic indicators, such as employment and industrial production, and financial indicators, such as stock prices and interest rates. Each time series of monthly indicators is transformed into three quarterly time series, each containing observations for the first, second or third month of the quarter. Hence, there can be missing observations at the end of some of these three time series, depending on the specific month of the quarter that we are in. We then include in the model only the quarterly series without missing observations at the moment in time that the forecast is formed, which addresses the ragged edge of the data.

We use Bayesian methods to estimate the resulting model, which expands in size as more monthly data on the quarter become available. Bayesian estimation facilitates providing shrinkage on estimates of a model that can be quite large, conveniently generates predictive densities and readily allows for stochastic volatility.

We provide results on the accuracy of the resulting nowcasts of realtime GDP growth in the USA from 1985 through 2011. Whereas most prior nowcasting research has focused on the accuracy of point forecasts of GDP growth, we consider both point and density forecasts. It turns out that in terms of point forecasts our proposal improves significantly on AR models and performs comparably with survey forecasts. In addition, it easily provides reliable density forecasts, for which the stochastic volatility specification is quite useful.

To place our proposed approach within the broader nowcasting literature, it is helpful to use the ‘partial model’ (or single‐equation) methods and ‘full system’ methods classifications that were used by Banbura *et al*. (2013). The former type of approach involves specifications focused on the low frequency variable, in which the high frequency explanatory variables are not modelled. In the latter approach, the low and high frequency variables are jointly modelled. Our proposed modelling approach falls in the partial models class.

Among partial model methods, bridge and mixed data sampling (MIDAS) regression models are most commonly used. Bridge models, which were considered in such studies as Baffigi *et al*. (2004), Diron (2008) and Bencivelli *et al*. (2012), relate the period *t* value of the quarterly variable of interest, such as GDP growth, to the period *t* quarterly average of key monthly indicators. The period *t* average of each monthly indicator is obtained with data that are available within the quarter and forecasts for other months of the quarter (obtained typically from an AR model for the monthly indicator). MIDAS‐based models, which were developed in Ghysels *et al*. (2004) for financial applications and applied to macroeconomic forecasting by, for example, Clements and Galvao (2008) and Guerin and Marcellino (2013), relate the period *t* value of the quarterly variable of interest to a constrained distributed lag of monthly or weekly or even daily data on the predictors of interest. The resulting model is then estimated by non‐linear least squares and used to forecast the variable of interest from constrained distributed lags of the available data. Foroni *et al*. (2015) propose the use of unconstrained distributed lags of the high frequency indicators: a specification labelled unrestricted MIDAS.

Full system methods for nowcasting include factor models and mixed frequency VAR models. We refer to the surveys in Banbura *et al*. (2013) and Foroni *et al*. (2013) for details and references. Here we mention only a few studies that are closely related to our proposal. These include Aastveit *et al*. (2014), which, in contrast with most of the nowcasting literature, focuses on density forecasts, Chiu *et al*. (2011), Ghysels (2012), and Schorfheide and Song (2013) and McCracken and Sekhposyan (2012), both of which developed mixed frequency Bayesian VAR models, and Marcellino *et al*. (2012), which introduced a small scale factor model that allows for stochastic volatility in the common and idiosyncratic components, and provided density forecasts.

Relative to the existing partial model and full system approaches, the innovations in our approach include the use of Bayesian shrinkage and the inclusion of stochastic volatility. Bayesian shrinkage often improves the accuracy of forecasts from time series models, and it permits us to include a potentially large set of indicators, which some evidence (e.g. De Mol *et al*. (2008)) suggests should permit our model to achieve forecast accuracy comparable with that of factor models (full system methods). The use of direct‐type estimation means that we do not need to model explicitly the conditioning variables. Moreover, with the univariate forecasting equation of our approach, we can easily allow for stochastic volatility, a feature that is important to the accuracy of density forecasts, mostly neglected so far in the nowcasting literature (we can also easily allow time varying regression coefficients). Finally, parameter time variation in the coefficients of the conditional mean can be also allowed, though we did not find it useful in the empirical application and therefore we do not discuss it explicitly (see Carriero *et al*. (2013) for details).

The ability to include tractably a large set of indicators and stochastic volatility in the model gives our approach some advantages over other approaches in the partial model and full system classes. For example, with MIDAS methods, it is computationally difficult in general to consider more than a few indicators, whereas with factor model methods it is computationally difficult to include stochastic volatility unless only a small set of variables is used. However, admittedly, these other approaches can be seen as having some advantages over our proposed approach. For example, with MIDAS methods it is feasible to handle a large mismatch in frequencies, e.g. daily–quarterly, whereas handling this with our model could be challenging (though still feasible), as it would generate a large number of additional regressors. Moreover, it could be feasible to design MIDAS models where the same non‐linear polynomial applies to different indicators, which would reduce the extent of the non‐linearity and make the model more easily estimable even with several indicators. In practice, this would be an alternative to our use of Bayesian shrinkage to reduce the curse of dimensionality.

The paper is structured as follows. Because data choices and availability play into our model specification choices, we first present the data in Section 2. Section 3 details our model and estimation method, and Section 4 introduces competing nowcasts. We then present results in Section 5. Finally, we provide some concluding remarks in Section 6.

The data that are analysed in the paper and the programs that were used to analyse them can be obtained from

http://wileyonlinelibrary.com/journal/rss-datasets

## 2 Data

We focus on current quarter forecasting of real GDP (or gross national product (GNP) for some of the sample) in realtime. Quarterly realtime data on GDP or GNP are taken from the Federal Reserve Bank of Philadelphia's ‘Real‐time data set for macroeconomists’ (RTDSM). For simplicity, hereafter ‘GDP’ refers to the output series, even though the measures are based on GNP and a fixed weight deflator for much of the sample.

To forecast GDP, we consider 12 monthly indicators which are broadly informative about economic and financial development, selected with some eye to timeliness: payroll employment, industrial production, real retail sales (nominal deflated by the consumer price index), housing starts, the Institute for Supply Management (ISM) index (overall) for manufacturing, the ISM index for supplier delivery times, the ISM index for orders, average weekly hours of production and supervisory workers, new claims for unemployment insurance, stock prices as measured by the Standard and Poor's 500 index, the 10‐year Treasury bond yield and the 3‐month Treasury bill rate.

In selecting the set of indicators, we did not engage in a broad search for best indicators or endeavour to make comparisons of these indicators with others that have been found to work well in some studies. Of course, there are a range of others that could be worth considering. For example, if one were producing forecasts in the middle of the month (rather than early in the month as we do), the Federal Reserve Bank of Philadelphia's business survey would be worth considering (as in such studies as Giannone *et al*. (2008)). Moreover, in future research, it might also be worth considering indicators reported at a weekly or daily frequency. Although our method can handle these higher frequencies, we focus our application on monthly indicators, in light of the finding by Banbura *et al*. (2013) that higher frequency information does not seem to be especially useful for nowcasting US GDP growth (except perhaps in a continuous monitoring context).

Of the variables that we do use, for those subject to significant revisions—payroll employment, industrial production, retail sales and housing starts—we use realtime data, obtained from the RTDSM (employment, industrial production and housing starts) or the Federal Reserve Bank of St Louis ‘Archival Federal Reserve economic data’ database (retail sales). For the consumer price index, we use the 1967 base year index that is available from the Bureau of Labor Statistics rather than a realtime series; Kozicki and Hoffman (2004) showed that the 1967 base year series is very similar to realtime consumer price index inflation. For the other variables, subject to either small revisions or no revision, we simply use the currently available time series, obtained from the Federal Reserve Board's ‘Forecasting analysis and modeling environment’ database.

The full forecast evaluation period runs from 1985, quarter 1, through to 2011, quarter 3 (using period *t* to refer to a forecast for period *t*), which involves realtime data vintages from January 1985 through March 2012. For each forecast origin *t* starting in the first month of 1985, quarter 1, we use the realtime data vintage *t* to estimate the forecast models and construct forecasts of GDP growth in the quarter. In forming the data set that is used to estimate the forecasting models at each point in time, we use the monthly vintages of (quarterly) GDP that are available from the RTDSM, taking care to make sure that the GDP time series used in the regression is the time series that is available at the time that the forecast is being formed. The starting point of the model estimation sample is always 1970, quarter 2, the soonest possible given data availability and lags allowed in models.

In light of the potential for the large surprises of the recent sharp recession to alter results, we also report results for a sample ending in 2008, quarter 2, before the recession became dramatic.

Throughout the analysis, we shall focus on current quarter forecasts (corresponding to one‐step‐ahead forecasts for most of our models). Our method can easily be extended to longer forecast horizons, and we have generated results for horizons of two and four quarters ahead, but we found very little evidence of predictability at these longer horizons, in line with the nowcasting literature. Moreover, we should mention that, to be technically proper, our method requires independent and identically distributed (IID) errors, which is a hypothesis that could be violated when forecasting more than one quarter ahead. (However, in direct multistep models with constant volatilities, some other studies have applied Bayesian estimation methods, abstracting from the serial correlation that is created by overlapping forecast errors (e.g. Koop (2013)).)

As discussed in such sources as Croushore (2006) Romer and Romer (2000) and Sims (2002), evaluating the accuracy of realtime forecasts requires a difficult decision on what to take as the actual data in calculating forecast errors. The GDP data that are available today for, say, 1985, represent the best available estimates of output in 1985. However, output as defined and measured today is quite different from output as defined and measured in 1970. For example, today we have available chain‐weighted GDP; in the 1980s, output was measured with fixed weight GNP. Forecasters in 1985 could not have foreseen such changes and the potential effect on measured output. Accordingly, we follow studies such as Clark (2011), Faust and Wright (2009) and Romer and Romer (2000) and use the second available estimates in the quarterly vintages of the RTDSM of GDP or GNP as actuals in evaluating forecast accuracy. We have also computed results by using the first estimate of GDP and obtained qualitatively very similar results.

## 3 The Bayesian mixed frequency model with stochastic volatility

This section details our proposed nowcasting models. To help the discussion to flow, we first specify the general model forms in Section 3.1 and then in Section 3.2 detail the sets of indicators in the model. We conclude by presenting in Sections 3.3 and 3.4 the priors and algorithms that are used in estimation.

### 3.1 General model forms

Starting with our specification that treats the error variance of the model as constant over time, we consider nowcasting the quarterly growth rate of GDP in month *m* of the current quarter based on the regression

(1) $$y_{t}=X_{m,\;t}^{\prime }\beta_{m}+v_{m,\;t},v_{m,\;t}\overset{\mathrm{IID}}{∼}N\left(0,\sigma_{m}^{2}\right),$$

where the vector $X_{m,\;t}$ contains the available predictors at the time that the forecast is formed, *t* is measured in quarters and *m* indicates a month within the quarter. As detailed below, given a set of monthly indicators to be used, there is a different regressor set $X_{m,\;t}$ (and therefore model) for each month *m* within the quarter, reflecting data availability.

In the stochastic volatility case, our proposed forecasting model for month *m* within the quarter takes the form

(2) $$\begin{array}{cc} & y_{t}=X_{m,\;t}^{\prime }\beta_{m}+v_{m,\;t},\\ & v_{m,\;t}=\lambda_{m,\;t}^{0.5}\varepsilon_{m,\;t},\varepsilon_{m,\;t}\overset{\mathrm{IID}}{∼}N\left(0,1\right),\\ & \log\left(\lambda_{m,\;t}\right)=\log\left(\lambda_{m,\;t-1}\right)+\nu_{m,\;t},\nu_{m,\;t}\overset{\mathrm{IID}}{∼}N\left(0,\phi_{m}\right). \end{array}$$

Following the approach that was pioneered in Cogley and Sargent (2005) and Primiceri (2005), the logarithm of the conditional variance of the error term in equation (2) follows a random‐walk process (in unreported results, we found that treating log‐volatility as an AR(1) process with a coefficient of 0.9 slightly reduced the forecast accuracy). In a VAR context, studies such as Clark (2011), Carriero *et al*. (2012) and D’Agostino *et al*. (2013) have found that this type of stochastic volatility formulation improves the accuracy of both point and density forecasts.

The specification of the regressor vector $X_{m,\;t}$ in the BMF and BMFSV models is partly a function of the way that we sample the monthly variables. For each monthly variable, we first transform it at the monthly frequency as necessary to achieve stationarity. At the quarterly frequency, for each monthly variable, we then define three different variables, by sampling the monthly series separately for each month of the quarter.

Exactly what variables are included in $X_{m,\;t}$ depends on when in the quarter the forecast is formed. We consider four timings for forecasting period *t* GDP growth: forecasting at the end of the first week of month 1 of quarter *t* (*m*=1), at the end of the first week of month 2 of quarter *t* (*m*=2), at the end of the first week of month 3 (*m*=3) and at the end of the first week of month 1 of quarter *t*+1 (*m*=4). These points in time are chosen to correspond to the usual timing of the publication of employment data: employment data for month *s* are normally published at the end of the first week of month *s*+1.

At each of the four forecast origins that we consider, for each quarter *t*, the regressor set $X_{m,\;t}$ is specified to include the subset of variables that are available for *t* (details are given in Section 3.2). At these points in time, the availability of other indicators also varies. As a consequence, the model specification changes in each month of the quarter, reflecting and accommodating the ragged edge of the data, in line with a direct approach to forecasting.

We stress that this approach does not involve bridge methods. Bridge methods require forecasting monthly observations of monthly variables for any months of quarter *t* for which data are not yet available. We do not use such forecasts. Rather, on the right‐hand side of the regression model we put only the actual monthly observations that are available for the quarter, in the form of different quarterly variables associated with the different months of the quarter.

### 3.2 Indicators used

We report below results for both ‘large’ and ‘small’ versions of the BMF and BMFSV models. The large version includes a broad set of 12 monthly indicators: payroll employment (log‐change); industrial production (log‐change); real retail sales (log‐change); housing starts (logarithmic); the ISM index (overall) for manufacturing; the ISM index for supplier delivery times; the ISM index for orders; average weekly hours of production and supervisory workers (log‐change); new claims for unemployment insurance; stock prices as measured by the Standard and Poor's 500 index (log‐change); the 10‐year Treasury bond yield; the 3‐month Treasury bill rate. The small version uses just the first five indicators of the 12‐variable set, which might be considered primary contemporaneous indicators of economic activity. In the results that are reported in this paper, in the model we include only values of these variables for the current quarter *t* (the quarter for which GDP growth is being forecast). However, our general approach easily allows the use of values from previous quarters (although this makes the models even larger, Bayesian shrinkage helps to limit the effects of parameter estimation error on forecast accuracy). Indeed, in Carriero *et al*. (2013) we also reported results for models in which the period *t*−1 (previous quarter) values of every variable are also included as a predictor.

Both the large and the small model specifications all include in $X_{m,\;t}$ a constant and one lag of GDP growth. In most cases, this means that the models include GDP growth in period *t*−1. However, in the case of models for forecasting at the end of the first week of month 1 of quarter *t*, the value of GDP growth in period *t*−1 is not available in realtime. In this case, the model includes GDP growth in period *t*−2. This is consistent with our general direct multistep specification of the forecasting models.

As noted above, depending on the month of the quarter the forecast is being formed, exactly what variables are in the large and small BMF and BMFSV models (i.e. in $X_{m,\;t}$) varies. Table 1 details the model specifications (and variable timing) that we use, based on the usual publication schedules of the indicators. Consider, for example, the version of the model that is used to forecast GDP growth at month *m*= 2 of the quarter. In this case, reflecting data availability, the small BMF and BMFSV models include in $X_{m,\;t}$ the following variables: a constant, GDP growth in quarter *t*−1 and employment growth and the ISM index in month 1 of quarter *t*. At month *m*= 3 of the quarter, with more data available, the small BMF and BMFSV models include in $X_{m,\;t}$ the following variables: a constant, GDP growth in quarter *t*−1, employment growth and the ISM index in month 2 of quarter *t*, and employment growth, the ISM, growth in industrial production, growth in retail sales, and log‐housing‐starts in month 1 of quarter *t*.

**Table 1:** Specifications of BMF models of GDP growth^a^

*Model*	*Predictors in model for the following months and quarters:*			
	*Month 1,*	*Month 2,*	*Month 3,*	*Month 1,*
	*quarter t*	*quarter t*	*quarter t*	*quarter t + 1*
1, AR	Two lags of GDP growth	Two lags of GDP growth	Two lags of GDP growth	Two lags of GDP growth
	estimated up to *t*−2	estimated up to *t*−1	estimated up to *t*−1	estimated up to *t*−1
2, small BMF and BMFSV	GDP (*t*−2)	GDP (*t*−1)	GDP (*t*−1)	GDP (*t*−1)
	emp (months 1–3 of *t*−1)	emp (month 1 of *t*)	emp (months 1 and 2 of *t*)	emp (months 1–3 of *t*)
	ISM (months 1–3 of *t*−1)	ISM (month 1 of *t*)	ISM (months 1 and 2 of *t*)	ISM (months 1–3 of *t*)
	IP (months 1 and 2 of *t*−1)		IP (month 1 of *t*)	IP (months 1 and 2 of *t*)
	RS (months 1 and 2 of *t*−1)		RS (month 1 of *t*)	RS (months 1 and 2 of *t*)
	starts (months 1 and 2 of *t*−1)		starts (month 1 of *t*)	starts (months 1 and 2 of *t*)
3, large BMF and BMFSV	GDP (*t* – 2)	GDP (*t* – 1)	GDP (*t* – 1)	GDP (*t* – 1)
	emp (months 1–3 of *t*−1)	emp (month 1 of *t*)	emp (months 1 and 2 of t)	emp (months 1–3 of *t*)
	ISM (months 1–3 of *t*−1)	ISM (month 1 of *t*)	ISM (months 1 and 2 of t)	ISM (months 1–3 of *t*)
	IP (months 1 and 2 of *t*−1)	supdel (month 1 of *t*)	IP (month 1 of *t*)	IP (months 1 and 2 of *t*)
	RS (months 1 and 2 of *t*−1)	orders (month 1 of *t*)	RS (month 1 of *t*)	RS (months 1 and 2 of *t*)
	starts (months 1 and 2 of *t*−1)	hours (month 1 of *t*)	starts (month 1 of t)	starts (months 1 and 2 of *t*)
	supdel (months 1–3 of *t*−1)	stprice (month 1 of *t*)	supdel (months 1 and 2 of *t*)	supdel (months 1–3 of *t*)
	orders (months 1–3 of *t*−1)	tbill (month 1 of *t*)	orders (months 1 and 2 of *t*)	orders (months 1–3 of *t*)
	hours (months 1–3 of *t*−1)	tbond (month 1 of *t*)	hours (months 1 and 2 of *t*)	hours (months 1–3 of *t*)
	claims (months 1 and 2 of *t*−1)		claims (month 1 of *t*)	claims (months 1 and 2 of *t*)
	stprice (months 1–3 of *t*−1)		stprice (months 1 and 2 of *t*)	stprice (months 1–3 of *t*)
	tbill (months 1–3 of *t*−1)		tbill (months 1 and 2 of *t*)	tbill (months 1–3 of *t*)
	tbond (months 1–3 of *t*−1)		tbond (months 1 and 2 of *t*)	tbond (months 1–3 of *t*)

a All models include a constant. Variables are defined as follows: employment, emp; ISM manufacturing index, ISM; industrial production, IP; retail sales, RS; housing starts, starts; ISM index of supplier delivery times, supdel; ISM index of new orders, orders; average weekly hours worked, hours; new claims for unemployment insurance, claims; Standard and Poor's index of stock prices, stprice; 3‐month Treasury bill rate, tbill; 10‐year Treasury bond, tbond. The variable transformations are given in Section 3.

Among these specifications, the largest empirical model that we consider includes 34 explanatory variables in $X_{m,\;t}$ (including up to 3 months of observations within the quarter for 12 monthly indicators, a constant and a lag of GDP—see Table 1 for the precise list). Although the ‘small’ BMF model is relatively small, it is not small in an absolute sense: depending on the month of the quarter, the model includes in $X_{m,\;t}$ up to 14 regressors (including up to 3 months of observations within the quarter for the five monthly indicators, a constant and a lag of GDP growth—see Table 1 for the precise list). With models of these sizes, under simple ordinary least squares (OLS) estimation, parameter estimation error would have large adverse effects on forecast accuracy. Our Bayesian approach to estimation incorporates shrinkage to help to limit the effects of parameter estimation error on forecast accuracy. We ran some checks with some of our basic models to verify the importance of this shrinkage to nowcast accuracy. These checks showed that models without shrinkage yielded root‐mean‐squared errors (RMSEs) that were 14–26% higher and average log‐scores that were 9–21% lower than the same models estimated with shrinkage (specifically, with the prior settings that are described in Section 3.3).

### 3.3 Priors

We estimate the models with constant volatility by using a normal–diffuse prior. As detailed in sources such as Kadiyala and Karlsson (1997), this prior combines a normal distribution for the prior on the regression coefficients with a diffuse prior on the error variance of the regression. For the models with stochastic volatility, we use independent priors for the coefficients (normal distribution) and volatility components (details are given below). Since the form of the prior is not dependent on *m*, in spelling out the prior we drop the index *m* from the model parameters for notational simplicity.

In all cases, for the coefficient vector *β*, we use a prior distribution that is normal, with mean 0 (for all coefficients) and variance that takes a diagonal, Minnesota style form. The prior variance is Minnesota style in the sense that shrinkage increases with the lag (with the quarter; not with the month within the quarter) and, in the sense that we impose more shrinkage on the monthly predictors than on lags of GDP growth (for the small BMF model, loosening up the cross‐variable shrinkage did not improve the results). The shrinkage is controlled by three hyperparameters (in all cases, a smaller number means more shrinkage): $\lambda_{1}$, which controls the overall rate of shrinkage, $\lambda_{2}$, which controls the rate of shrinkage on the monthly predictors relative to the shrinkage on the lags of GDP growth, and $\lambda_{3}$, which determines the rate of shrinkage that is associated with longer lags.

At each forecast origin, the prior standard deviation that is associated with the coefficient on variable $x_{i,\;j,\;t-l}$ of $X_{m,\;t}$, where *i* denotes the indicator (employment, etc.), *j* denotes the month within the quarter at which the indicator has been sampled and *l* denotes the lag in quarters (whereas we consider a lag of only 1 in this paper, Carriero *et al*. (2013) included results for models with a lag of 2), is specified as follows:

(3) $${\text{sd}}_{i,\;j,\;t-l}=\frac{\sigma_{\mathrm{GDP}}}{\sigma_{i,\;j}}\frac{\lambda_{1}\lambda_{2}}{l^{\lambda_{3}}}.$$

For coefficients on lag *l* of GDP, the prior standard deviation is

(4) $${\text{sd}}_{l}=\lambda_{1}/l^{\lambda_{3}}.$$

Finally, for the intercept, the prior is uninformative:

(5) $${\text{sd}}_{\mathrm{int}}=1000\sigma_{\mathrm{GDP}}.$$

In setting these components of the prior, for $\sigma_{\mathrm{GDP}}$ and $\sigma_{i,\;j}$ we use standard deviations from AR(4) models for GDP growth and $x_{i,\;j,\;t}$ estimated with the available sample of data.

In all our results, the hyperparameters are set at values that may be considered very common in Minnesota‐type priors (see, for example, Litterman (1986)): $\lambda_{1}=0.2$, $\lambda_{2}=0.2$ and $\lambda_{3}=1$. In Carriero *et al*. (2013), we ran some limited checks (for BMF models with different subsets of our 12 monthly indicators) to see what hyperparameter settings would be optimal in a realtime RMSE minimizing sense. To simplify the optimization, we focused on just $\lambda_{2}$. In effect, the parameter $\lambda_{1}$ can be seen as pinning down the rate of shrinkage for the lags of GDP growth in the model, whereas, given $\lambda_{1}$, $\lambda_{2}$ pins down the rate of shrinkage on the coefficients of the monthly indicators. Specifically, after simply fixing $\lambda_{1}$ at a conventional value of 0.2, we specified a wide grid of values for $\lambda_{2}$ and generated time series of forecasts for each corresponding model estimate (for a limited set of models). We then looked at choosing $\lambda_{2}$ in pseudorealtime to minimize the RMSE of past forecasts, using 5‐ or 10‐year windows. For example, using a model with nine economic indicators and both current quarter and past quarter values of the indicators in the model, at the first evaluation point, in late 1989, the optimal $\lambda_{2}$ was 0.2. As forecasting moved forwards in time, the optimal setting drifted up a little and then down a little, before ending the sample at values as high as 1. For simplicity, in all the results in the paper, we leave $\lambda_{2}$ at 0.2 through all our analysis. It is possible that the more computationally intensive approach of optimizing shrinkage at each forecast origin could improve forecast accuracy but, in a VAR context, Carriero *et al*. (2014) found that the pay‐off to optimization over fixed, conventional shrinkage was small.

Finally, in the prior for the volatility‐related components of the model, our approach is similar to that used in such studies as Clark (2011), Cogley and Sargent (2005) and Primiceri (2005). For the prior on *ϕ*, we use a mean of 0.035 and 5 degrees of freedom. For the period 0 value of volatility of each equation *i*, we use a prior of

(6) $$\begin{array}{cc} & \mu_{\lambda }=\log\left({\hat{\lambda }}_{0,\;\mathrm{OLS}}\right),\\ & \Omega_{\lambda }=4. \end{array}$$

To obtain $\log({\hat{\lambda }}_{0,\;\mathrm{OLS}})$, we use a training sample of 40 observations preceding the estimation sample to fit an AR(4) model to GDP growth.

### 3.4 Estimation algorithms

The model with constant volatility is estimated with a Gibbs sampler, using the approach for the normal–diffuse prior and posterior detailed in such studies as Kadiyala and Karlsson (1997). At any given forecast origin, estimation is quite fast, because the forecasting model is a single equation.

The model with stochastic volatility is estimated with a Metropolis‐within‐Gibbs algorithm, used in such studies as Clark (2011) and Carriero *et al*. (2012). The posterior mean and variance of the coefficient vector are given by

(7) $${\overset{¯}{\mu }}_{\beta }={\overset{¯}{\Omega }}_{\beta }(\sum_{t=1}^{T}\lambda_{t}^{-1}X_{m,\;t}y_{t}+\Omega_{\beta }^{-1}\mu_{\beta }),$$

(8) $${\overset{¯}{\Omega }}_{\beta }^{-1}=\Omega_{\beta }^{-1}+\sum_{t=1}^{T}\lambda_{t}^{-1}X_{m,\;t}X_{m,\;t}^{\prime },$$

where we again omit the *m*‐index from the parameters for notational simplicity.

In presenting our results, we focus on forecasts that are obtained by estimating the forecasting models with a recursive scheme: the estimation sample expands as forecasting moves forwards in time. A rolling scheme, under which the size of the estimation sample remains fixed over time but the first observation moves forwards in time, is in general less efficient but can be more robust in the presence of changes in regression parameters and (for density forecasts) error variances. Hence, for the BMF models with constant volatility we also report results based on a rolling estimation scheme. However, as we shall show below, rolling window estimation of the model is not sufficient to obtain point and density forecasts that are as good as those obtained with the (recursive) BMFSV specification (with a gap that is particularly large for density forecasts).

In all cases, we obtain forecast distributions by sampling as appropriate from the posterior distribution. For example, in the case of the BMFSV model, for each set of draws of parameters, we

- simulate volatility for the quarter being forecast by using the random‐walk structure of log‐volatility,
- draw shocks to the variable with variance equal to the draw of volatility and
- use the structure of the model to obtain a draw of the future value (i.e. forecast) of the variable.

We then form point forecasts as means of the draws of simulated forecasts and density forecasts from the simulated distribution of forecasts. Conditionally on the model, the posterior distribution reflects all sources of uncertainty (latent states, parameters and shocks over the forecast interval).

## 4 Competing nowcasts

We compare our BMF and BMFSV nowcasts with those generated from AR models and with survey‐based forecasts (which pool many predictions, based on timely information). These are typically tough benchmarks in forecast competitions. The results in Carriero *et al*. (2013) also indicate that MIDAS and unrestricted MIDAS specifications can produce relatively good nowcasts of GDP growth. However, these models are primarily designed for use with small sets of indicators (possibly with a high frequency mismatch) and point forecasts; using large sets of indicators and allowing stochastic volatility to obtain reliable density forecasts is feasible but relatively difficult. As a result, we abstract from MIDAS and unrestricted MIDAS forecasts in the comparison.

### 4.1 Auto‐regressive models

In our forecast evaluation, in light of evidence in other studies of the difficulty of beating simple AR models for GDP growth, we include forecasts from AR(2) models. The models take the same basic forms given in expressions (1) and (2), with $X_{m,\;t}$ defined to include just a constant and two lags of GDP growth. In keeping with our realtime set‐up, we generate four different AR‐based forecasts of GDP growth in each quarter *t*, based on the data that are available in realtime at the end of the first week of month 1 of quarter *t*, at the end of the first week of month 2 of quarter *t*, at the end of the first week of month 3 and at the end of the first week of month 1 of quarter *t*+1. The models based on month 2, month 3 and month 1 of quarter *t*+1 are all conventional AR(2) specifications relating GDP in quarter *t* to GDP in quarters *t*−1 and *t*−2. For a given quarter, these model estimates and forecasts differ only in that the GDP data that are available for estimation and forecasting will differ across the months and data vintages. However, the specification of the model based on month 1 of quarter *t* differs, because GDP growth for period *t*−1 is not yet available. In this case, the model takes a direct multistep form, relating GDP in quarter *t* to GDP in quarters *t*−2 and *t*−3, and the forecast horizon is in effect two quarters, not one quarter. In all cases, in light of prior evidence of the success of AR models estimated by least squares, we estimate the AR models with extremely loose priors, so that our Bayesian estimates based on the normal–diffuse prior effectively correspond to least squares estimates.

### 4.2 Surveys

We also consider GDP growth nowcasts based on the Survey of Professional Forecasters (SPF), which is available quarterly, and the Blue Chip (BC) Consensus, which is available monthly, since they are closely monitored by decision makers and typically perform quite well. The forecasts from the nowcasting models, BC, and the SPF reflect information sets that, in terms of timing, should be similar. In particular, the BC survey is conducted a few days before publication on the 10th of each month. So it should usually be the case that BC respondents have available the same information as each nowcasting model uses. For example, for month 2 of quarter *t*, we define the model to use information that is normally available at the end of the first week of the month, which will include employment and the ISM for month 1 of the quarter. At the time of the BC survey, that same information would normally be available to participating forecasters. In the case of the SPF forecast, the mid‐quarter timing of the survey means that the SPF forecast should be comparable with only the BC and model forecasts that are made in month 2 of the quarter (although most comparable, the SPF forecast should normally reflect a little more information than would be available to the BC survey or the models).

## 5 Results

This section presents results on the accuracy of point and density forecasts from our proposed BMF and BMFSV methods relative to the accuracy of forecasts from AR models, the SPF and BC survey. For the SPF and BC forecasts, our comparisons are limited to point forecasts. The section first describes the metrics that were used and then provides the results. As noted in Section 2, we present results for both a full sample of 1985, quarter 1–2011, quarter 3, and a precrisis sample of 1985, quarter 1–2008, quarter 2.

### 5.1 Metrics

To assess the accuracy of point forecasts, we use RMSEs. To facilitate the presentation, we report RMSEs for each nowcasting model, BC and SPF relative to the AR model with constant volatility. To provide a rough gauge of whether the differences in RMSEs are statistically significant, we use the Diebold and Mariano (1995)–West (1996) *t*‐statistic for equal MSE, applied to the forecast of each model relative to the benchmark.

For comparing our proposed BMF and BMFSV forecasts with AR model forecasts, the overlap between each alternative model and the benchmark could in principle complicate inference. Our models of interest do not strictly nest the AR models, because the AR models include two lags of GDP growth whereas the nowcasting models include just one lag of GDP growth. But it is possible that the models overlap, in the sense that the true model could be an AR(1) specification. However, since forecast performance suggests that it is unlikely that the AR model and nowcasting models overlap, we proceed to treat them as being non‐nested. The results in West (1996) imply that we can test equal accuracy of point forecasts from non‐nested models by computing a simple *t*‐test for equal MSE, as we do. To capture some low order serial correlation, we compute the *t*‐statistics with a heteroscedasticity and auto‐correlation consistent variance, using a rectangular kernel and bandwidth of 1 and the small sample adjustment of Harvey *et al*. (1997).

To assess the accuracy of density forecasts, we use log‐predictive‐density scores, motivated and described in such sources as Geweke and Amisano (2010). At each forecast origin, we compute the log‐predictive‐score by using the realtime outcome and the probability density of the forecast. For all models, we compute the density by using an empirical estimate of the forecast density based on 5000 draws of forecasts, a non‐parametric density estimator and a Gaussian kernel. To facilitate model comparisons, we report average log‐scores for our BMF and BMFSV models relative to a benchmark AR model with stochastic volatility (ARSV). To provide a rough gauge of the statistical significance of differences in average log‐scores, we use the Amisano and Giacomini (2007) *t*‐test of equal means, applied to the log‐score for each model relative to the ARSV model. We view the tests as a rough gauge because, for forecasts from estimated models, the asymptotic validity of the Amisano and Giacomini (2007) test requires that, as forecasting moves forwards in time, the models be estimated with a rolling, rather than expanding, sample of data. To allow for the potential of some serial correlation in score differences, we compute the *t*‐statistics with a heteroscedasticity and auto‐correlation consistent variance estimate obtained with a rectangular kernel and bandwidth of 1.

As further checks on density forecast calibration, we also provide results on the accuracy of interval forecasts and selected results for probability integral transforms (PITs). Motivated in part by central bank interest in forecast confidence intervals and fan charts, recent studies such as Giordani and Villani (2010) have used interval forecasts as a measure of forecast accuracy for macroeconomic density forecasts. We compute results for 70% interval forecasts, defined as the frequency with which realtime outcomes for GDP growth fall inside 70% highest posterior density intervals estimated in realtime for each model. To provide a rough gauge of statistical significance, we include *p*‐values for the null hypothesis of correct coverage (empirical = nominal rate of 70%), based on *t*‐statistics computed with a heteroscedasticity and auto‐correlation consistent variance estimate obtained with a rectangular kernel and bandwidth of 1. The *p*‐values provide only a rough gauge of significance in the sense that the theory underlying Christofferson's (1998) test results abstracts from possible effects of forecast model estimation—i.e. parameter estimation error.

The PIT that was emphasized by Diebold *et al*. (1998) provides a more general indicator of the accuracy of density intervals than does an interval forecast coverage rate. For an illustrative set of models, we provide PIT histograms, obtained as decile counts of PIT transforms. For optimal density forecasts at the one‐step horizon, the PIT series would be independent uniform(0,1) random variables. Accordingly, the histograms would be flat. To provide some measure of the importance of departures from the IID uniform distribution, we include in the histograms 90% intervals estimated under the binomial distribution (following Diebold *et al*. (1998)). These intervals are intended to be only a rough guide to significance of departures from uniformity; more formal testing would require a joint test (for all histogram bins) and addressing the possible effects of model parameter estimation on the large sample distributions of PITs.

### 5.2 Point forecasts

To assess the accuracy of point forecasts, Table 2 provides RMSE comparisons of our proposed BMF and BMFSV nowcasting models, BC survey and the SPF to forecasts from the AR model. To facilitate comparisons, the first row of each part of Table 2 provides the RMSE of the AR model forecast (as noted above, the RMSEs of the AR model can change across months of the quarter, because of a change in the model specification from the first month of the quarter to the second and to GDP data revisions from month to month). The remaining rows provide the ratio of each forecast's RMSE relative to the AR model's RMSE. A number less than 1 means that a given forecast is more accurate than the AR model. The numbers in parentheses are the *p*‐values of two‐sided *t*‐statistics for equal MSE. The two panels of Table 2 refer to the periods 1985, quarter 1–2011, quarter 3, and 1985, quarter 1–2008, quarter 2.

**Table 2:** RMSEs relative to the AR model benchmark^a^

*Forecast*	*Results for the following months and quarters:*			
	*Month 1,*	*Month 2,*	*Month 3,*	*Month 1,*
	*quarter t*	*quarter t*	*quarter t*	*quarter t + 1*
*1985, quarter 1–2011, quarter 3*				
AR	2.213	2.066	2.046	2.029
BC	0.831 (0.251)	0.845 (0.305)	0.757 (0.133)	0.622 (0.034)
SPF	—b	0.803 (0.206)	—b	—b
ARSV	0.999 (0.922)	1.007 (0.227)	1.007 (0.342)	1.010 (0.191)
Small BMF	0.932 (0.167)	0.916 (0.102)	0.823 (0.120)	0.770 (0.091)
Large BMF	0.932 (0.210)	0.872 (0.141)	0.800 (0.207)	0.770 (0.175)
Small BMF, rolling	1.000 (0.989)	0.984 (0.120)	0.890 (0.064)	0.838 (0.053)
Large BMF, rolling	1.002 (0.966)	0.952 (0.079)	0.903 (0.322)	0.850 (0.241)
Small BMFSV	0.936 (0.248)	0.906 (0.098)	0.815 (0.108)	0.749 (0.071)
Large BMFSV	0.925 (0.351)	0.879 (0.234)	0.816 (0.246)	0.771 (0.173)
*1985, quarter 1–2008, quarter 2*				
AR	1.820	1.758	1.745	1.733
BC	0.963 (0.665)	0.983 (0.824)	0.881 (0.123)	0.737 (0.001)
SPF	—b	0.930 (0.331)	—b	—b
ARSV	1.002 (0.829)	1.005 (0.498)	1.010 (0.312)	1.010 (0.309)
Small BMF	0.960 (0.050)	0.941 (0.015)	0.859 (0.018)	0.816 (0.005)
Large BMF	0.979 (0.567)	0.914 (0.024)	0.892 (0.144)	0.864 (0.062)
Small BMF, rolling	1.003 (0.859)	0.981 (0.196)	0.890 (0.027)	0.830 (0.004)
Large BMF, rolling	0.992 (0.692)	0.950 (0.068)	0.920 (0.194)	0.867 (0.041)
Small BMFSV	0.955 (0.159)	0.926 (0.045)	0.852 (0.025)	0.799 (0.003)
Large BMFSV	1.000 (0.995)	0.941 (0.292)	0.914 (0.268)	0.870 (0.070)

a RMSE for AR, RMSE ratios for all others; *p*‐values of equal MSEs are given in parentheses. See Table 1 and Sections 3 and 3.1 for the definition of the models. The equal forecast accuracy test is described in Section 5.1. The reported RMSEs reflect GDP growth defined in annualized percentage terms.

b Not applicable.

We can draw six main conclusions from the RMSE results in Table 2. First, as might be expected, the accuracy of forecasts from the BMF and BMFSV models improves as more data on the quarter become available, and we move from month 1 to 2, 2 to 3, and 3 to month 1 of the next quarter. The gains look a little bigger with the move from month 2 to month 3 than from month 3 to month 1 of the next quarter. As a consequence, the accuracy of the nowcasting models relative to the AR baseline increases with the addition of more information on the quarter. For both the small and the large BMF and BMFSV models, the gain in RMSE in the full sample results rises from about 7% in month 1 to 18% in month 3 and 23–25% in month 1 of the next quarter. Somewhat surprisingly, the Diebold–Mariano–West test does not often imply that the gains are statistically significant in the full sample, but it does imply more significance in the sample that ends before the depths of the crisis.

Second, in the sample that ends in mid‐2008 and thereby avoids the huge forecast errors of the severe recession, our BMF and BMFSV nowcasting models are often as accurate as or even a little more accurate (although not significantly so) than those of the BC survey, particularly in months 2 and 3 of quarter *t*. However, from month 3 of quarter *t* to month 1 of quarter *t*+1, the BC forecasts improve in accuracy more so than do the model forecasts. As a result, in month 1 of quarter *t*+1, the nowcasting models are generally less accurate than the BC results although not dramatically so.

**Figure 1:**
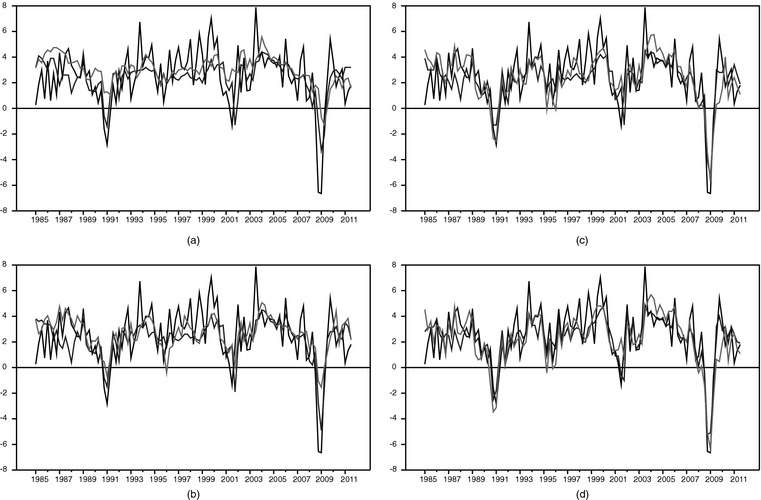
Realtime point forecasts of GDP growth, 1985 quarter 1–2011, quarter 3 (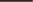, actual GDP; 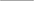, BC; 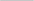, large BMFSV): (a) in month 1 of quarter *t*; (b) in month 2 of quarter *t*; (c) in month 3 of quarter *t*; (d) in month 1 of quarter *t*+1

To shed some further light on the performance of the nowcasting models and BC forecast over time, Fig. 1 compares actual quarterly GDP growth (annualized) to point forecasts from the BC survey and our large BMFSV nowcasting model. The chart makes clear the improvement in accuracy that occurs with the addition of more data on the quarter—improvement that seems most noticeable around recessions (1990–1991, 2001 and 2007–2009). It also shows that, over some periods of time, the model is more accurate than the BC forecast, whereas, in others, the BC forecast is more accurate than the model. One period in which the BC forecast fares better is the most recent recession, when it did a better job of picking up and projecting unprecedented declines in GDP growth.

Accordingly, the third main conclusion from the RMSE results is that, in the full sample, the nowcasting models are somewhat less accurate than the BC forecast, perhaps because of the relative performance in the depths of the crisis, though the differences are not statistically significant. The challenge of beating a survey forecast with good nowcasting models is also evident in such studies as Banbura *et al*. (2013), who developed a mixed frequency factor model‐based forecast that is comparable with, but not quite as good as, the SPF in forecasts for 1995–2010.

In light of the evidence in Chauvet and Potter (2013) that the advantage of some time series models over an AR model baseline stems largely from periods of recession, not during economic expansions (or normal times), we have checked the forecast performance of our models during just economic expansions (dropping out observations falling during National Bureau of Economic Research recessions). During expansions, our nowcasting models also forecast more accurately than the AR baseline. That said, in terms of RMSEs, the advantages of the nowcasting models over the AR baseline are somewhat smaller when recessions are excluded than in the full sample. (The expansion *versus* recession distinction is smaller in density forecast accuracy than in point forecast accuracy.) Overall, the advantages of our models over an AR baseline may be less affected by the expansion *versus* recession distinction than the models of Chauvet and Potter (2013) were affected because our models exploit more within‐the‐quarter indicators of economic activity.

Returning to the primary conclusions from our results, a fourth conclusion to draw from Table 2 is that including stochastic volatility in our proposed BMF nowcasting model does not have much pay‐off, or cost, in terms of the accuracy of point forecasts. Broadly, for a given variable set included in a nowcasting model, BMF and BMFSV yield similar RMSE ratios, with the stochastic volatility version sometimes a little better and other times a little worse.

Fifth, there are no major differences between the small (again, small is relative—even the small model involves as many as 14 regressors) and large BMF models. The former are slightly better when the sample ends in 2008; the latter over the full sample, suggesting that more information became relevant during the crisis. We have also experimented (in Carriero *et al*. (2013)) with different subgroups of the indicators. Perhaps the most interesting finding is that financial indicators, by themselves, do poorly in forecasting current quarter GDP growth. However, in line with the comparison of results across samples that was mentioned above, including financial indicators with other indicators helps the models (a little) during the recent crisis.

Finally, rolling estimation of the BMF models with constant volatilities generates systematically higher RMSEs than recursive estimation of the same models. This finding suggests that the efficiency losses from using a smaller set of observations are larger than the gains from achieving partial robustness to possible breaks.

The main message that we can take from the point forecast evaluation is that overall our BMF method is superior to AR model forecasts and comparable with survey forecasts, though the surveys performed a little better during the crisis. However, a major advantage of our approach is that it also easily delivers density and interval forecasts, and, as we shall now see, in this context the stochastic volatility specification that we adopt becomes quite relevant.

### 5.3 Density forecasts: average predictive scores

To assess the calibration of density forecasts, Table 3 provides average log‐score comparisons of our BMF and BMFSV nowcasting models, taking an ARSV model as the benchmark (since previous research has shown that stochastic volatility improves density accuracy of AR forecasts). To facilitate comparisons, the first row of each part of Table 3 provides the average log‐score of the ARSV forecast; the remaining rows provide the score of each other model forecast less the benchmark score. Entries that are greater than 0 mean that a given density forecast is more accurate (has a higher score) than the ARSV baseline. The numbers in parentheses are the *p*‐values of two‐sided *t*‐statistics for tests of equality of average log‐scores. The two panels of Table 3 refer to the periods 1985, quarter 1–2011, quarter 3, and 1985, quarter 1–2008, quarter 2.

**Table 3:** Average log‐scores relative to the ARSV model benchmark^a^

*Forecast*	*Results for the following months and quarters:*			
	*Month 1,*	*Month 2,*	*Month 3,*	*Month 1,*
	*quarter t*	*quarter t*	*quarter t*	*quarter t + 1*
*1985, quarter 1–2011, quarter 3*				
ARSV	−2.210	−2.144	−2.134	−2.123
AR	−0.245 (0.000)	−0.258 (0.000)	−0.264 (0.000)	−0.269 (0.000)
Small BMF	−0.177 (0.015)	−0.151 (0.009)	−0.007 (0.916)	0.045 (0.493)
Large BMF	−0.145 (0.031)	−0.091 (0.186)	0.070 (0.416)	0.094 (0.251)
Small BMF, rolling	−0.185 (0.039)	−0.119 (0.032)	0.019 (0.649)	0.081 (0.065)
Large BMF, rolling	−0.141 (0.020)	−0.087 (0.113)	0.027 (0.610)	0.085 (0.208)
Small BMFSV	0.018 (0.558)	0.085 (0.010)	0.195 (0.002)	0.279 (0.000)
Large BMFSV	0.127 (0.002)	0.126 (0.017)	0.182 (0.065)	0.227 (0.020)
*1985, quarter 1–2008, quarter 2*				
ARSV	−2.091	−2.049	−2.049	−2.047
AR	−0.307 (0.000)	−0.312 (0.000)	−0.310 (0.000)	−0.307 (0.000)
Small BMF	−0.251 (0.000)	−0.208 (0.000)	−0.055 (0.275)	−0.002 (0.962)
Large BMF	−0.216 (0.000)	−0.155 (0.002)	0.003 (0.959)	0.034 (0.535)
Small BMF, rolling	−0.125 (0.018)	−0.108 (0.020)	0.012 (0.796)	0.082 (0.083)
Large BMF, rolling	−0.106 (0.036)	−0.067 (0.141)	0.023 (0.629)	0.083 (0.087)
Small BMFSV	0.028 (0.229)	0.079 (0.022)	0.178 (0.002)	0.259 (0.000)
Large BMFSV	0.088 (0.018)	0.103 (0.029)	0.150 (0.024)	0.182 (0.019)

a Score for ARSV, differences in score for all others; *p*‐values of equal mean scores are given in parentheses. See Table 1 and Sections 3 and 3.1 for the definition of the models. The average log‐score and the equal forecast accuracy test are described in Section 5.1. The reported scores reflect GDP growth defined in annualized percentage terms.

The main findings are as follows. First, including stochastic volatility in a model considerably improves its average log‐score. This is true for both the AR model and our BMF nowcasting models. Consider, for example, the small BMF model in month 2 of quarter *t*. The constant volatility version of the model yields an average score that is 15.1% below the ARSV baseline, whereas the stochastic volatility version yields a score that is 8.5% above the baseline.

**Figure 2:**
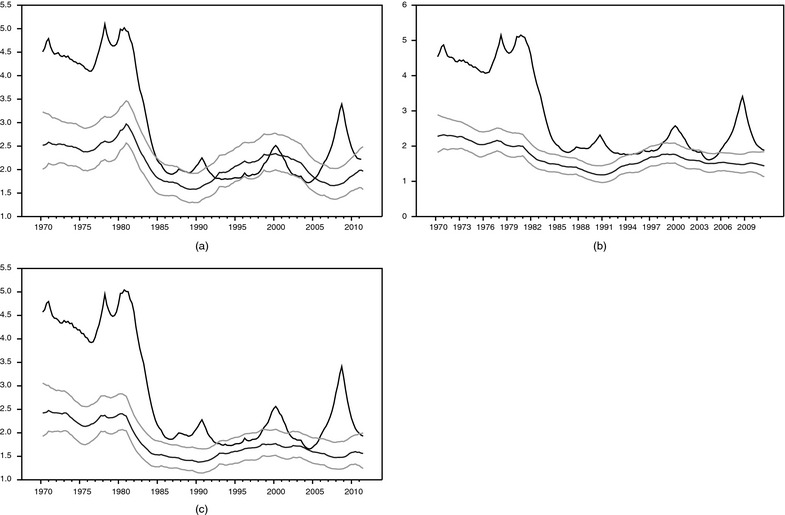
Volatility ($\lambda_{m,t}^{0.5}$ of equation (2)) (posterior medians of standard deviations) estimates from ARSV (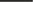) and large BMFSV (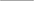) (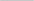, 15th and 85th percentiles) models, last vintage of data: (a) in month 2 of quarter *t*; (b) in month 3 of quarter *t*; (c) in month 1 of quarter *t*+1

To provide some intuition for the importance of allowing time varying volatility, Fig. 2 reports the estimates of stochastic volatility from an AR model and our large BMFSV nowcasting model, obtained from the full sample of data available in our last realtime data vintage. The volatility plotted is $\lambda_{m,\;t}^{0.5}$ from equation (2), *m*=1,2,3, corresponding to the standard deviation of shocks to GDP growth in each model. For the ARSV model, we report just the posterior median of $\lambda_{m,\;t}^{0.5}$; for the BMFSV model, we report the posterior median and the 70% credible set. The charts show that time variation in volatility is considerable for an AR model, reflecting the ‘Great Moderation’ and a rise in volatility during the recent recession. Including within‐quarter monthly indicators tends to dampen the swings in volatility, more so as more months of data within the quarter become available. However, even with the BMFSV nowcasting model, there continue to be sizable movements in volatility.

The second main finding is that the average log‐scores of the BMF and BMFSV models improve as more data become available for the quarter (i.e. scores are higher for models with 2 months of data than 1 month of data, etc.). As a consequence, some of the nowcasting models with 2 or 3 months of data on the quarter but constant volatility score better than the ARSV model. However, these gains are rarely statistically significant. Moreover, in the precrisis sample, nowcasting models with constant volatilities have a more difficult time beating the ARSV benchmark.

Third, both BMFSV models improve on the average log‐score of the baseline ARSV specification. The gains increase as the nowcasting models receive more months of data. In most cases, the gains are statistically significant, even in the case of month 1 of the quarter. The large model is better for short horizons; the small for longer horizons. However, results in Carriero *et al*. (2013) for different subgroups of the indicators indicate that financial indicators, by themselves (as opposed to in conjunction with other indicators, as in our large model), are not very helpful for density forecasting.

Finally, for density forecasting, rolling estimation of the BMF model with constant volatility sometimes (not always) improves on the accuracy of the recursively generated forecasts from the same model but falls short of the recursively estimated model with stochastic volatility. Consider forecasts from the small BMF model from month 3 of the quarter. Relative to the ARSV baseline, the recursively estimated BMFSV model has a score differential of 19.5%, compared with a score differential of 1.9% for the rolling window version of the BMF forecast and −0.7% for the recursive version of the BMF forecast. This finding suggests that, in alternative model formulations such as MIDAS, it would be necessary to incorporate stochastic volatility—which would be difficult to do given the non‐linear regression problem that is involved in MIDAS—to achieve material gains in density accuracy.

### 5.4 Interval forecasts

As another measure of density forecast accuracy, we consider interval forecasts. For all our econometric models, Table 4 provides coverage rates defined as the frequency with which actual GDP growth falls within 70% forecast intervals, along with *p*‐values for the test that empirical coverage equals the 70% nominal rate. A number greater or less than 70% means that a given model yields posterior density intervals that are, on average, respectively too wide or too narrow. The two panels of Table 4 refer to the periods 1985, quarter 1–2011, quarter 3, and 1985, quarter 1–2008, quarter 2.

**Table 4:** Coverage rates, nominal 70%^a^

*Forecast*	*Results for the following months and quarters:*			
	*Month 1,*	*Month 2,*	*Month 3,*	*Month 1,*
	*quarter t*	*quarter t*	*quarter t*	*quarter t + 1*
*1985, quarter 1–2011, quarter 3*				
AR	0.925 (0.000)	0.944 (0.000)	0.944 (0.000)	0.944 (0.000)
ARSV	0.720 (0.653)	0.692 (0.851)	0.720 (0.653)	0.729 (0.502)
Small BMF	0.925 (0.000)	0.935 (0.000)	0.925 (0.000)	0.916 (0.000)
Large BMF	0.925 (0.000)	0.925 (0.000)	0.869 (0.000)	0.897 (0.000)
Small BMF, rolling	0.822 (0.001)	0.841 (0.000)	0.813 (0.003)	0.813 (0.003)
Large BMF, rolling	0.794 (0.016)	0.841 (0.000)	0.785 (0.033)	0.766 (0.106)
Small BMFSV	0.748 (0.259)	0.729 (0.502)	0.748 (0.259)	0.757 (0.171)
Large BMFSV	0.673 (0.552)	0.673 (0.552)	0.626 (0.116)	0.720 (0.653)
*1985, quarter 1–2008, quarter 2*				
AR	0.947 (0.000)	0.957 (0.000)	0.968 (0.000)	0.957 (0.000)
ARSV	0.723 (0.614)	0.691 (0.859)	0.723 (0.614)	0.745 (0.323)
Small BMF	0.947 (0.000)	0.947 (0.000)	0.936 (0.000)	0.926 (0.000)
Large BMF	0.947 (0.000)	0.947 (0.000)	0.872 (0.000)	0.894 (0.000)
Small BMF, rolling	0.840 (0.000)	0.872 (0.000)	0.840 (0.000)	0.840 (0.000)
Large BMF, rolling	0.830 (0.001)	0.862 (0.000)	0.809 (0.008)	0.798 (0.019)
Small BMFSV	0.777 (0.076)	0.745 (0.323)	0.745 (0.323)	0.755 (0.215)
Large BMFSV	0.670 (0.541)	0.702 (0.964)	0.638 (0.216)	0.713 (0.786)

a *p*‐values of correct coverage are given in parentheses. See Table 1 and Sections 3 and 3.1 for the definition of the models. The coverage rate and the test of correct coverage are described in Section 5.1.

The coverage rates in Table 4 are striking. Recursive estimation of models with constant volatilities in all cases yields coverage rates of about 90%, which are in all cases significantly different from the nominal rate of 70%. Somewhat surprisingly, given the patterns in the score results, the coverage does not show much tendency to grow better with the addition of more data across months of the quarter (it does become a little better, but not much). This suggests that the improvement in predictive scores that occurs with the addition of months of data is due to improvement in the forecast mean.

However, estimating the same models (BMF with constant volatilities) with a rolling window of observations yields better coverage rates: as much as 10 percentage points lower than the rates that are obtained with recursively estimated models. But a simple rolling window approach is not enough to yield correct coverage: coverage rates for the rolling scheme versions of the BMF models are all (with one exception) statistically different from 70%.

Correct coverage is achieved by including stochastic volatility in our BMF specification. Our models with stochastic volatility in all cases yield coverage rates that are sufficiently close to 70% that they are not statistically different from 70% (at the 5% level of significance).

**Figure 3:**
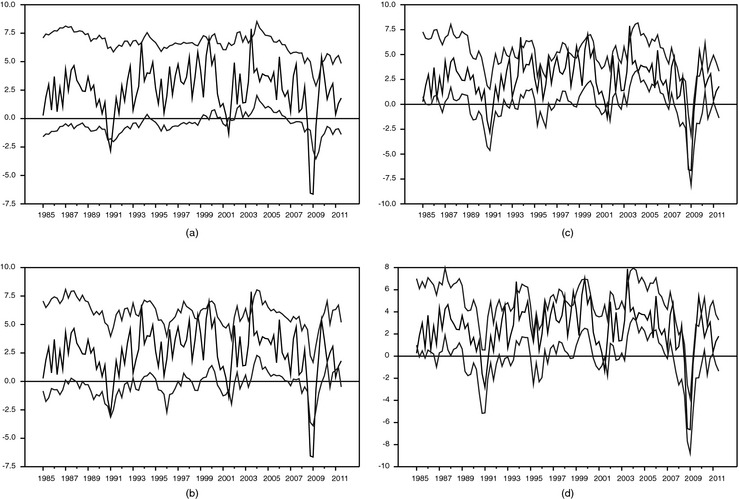
Realtime 70% interval forecasts of GDP growth from the large BMF model, 1985, quarter 1–2011, quarter 3 (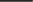, actual GDP; 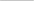, 15th and 85th percentiles): (a) in month 1 of quarter *t*; (b) in month 2 of quarter 1; (c) in month 3 of quarter *t*; (d) in month 1 of quarter *t*+1

**Figure 4:**
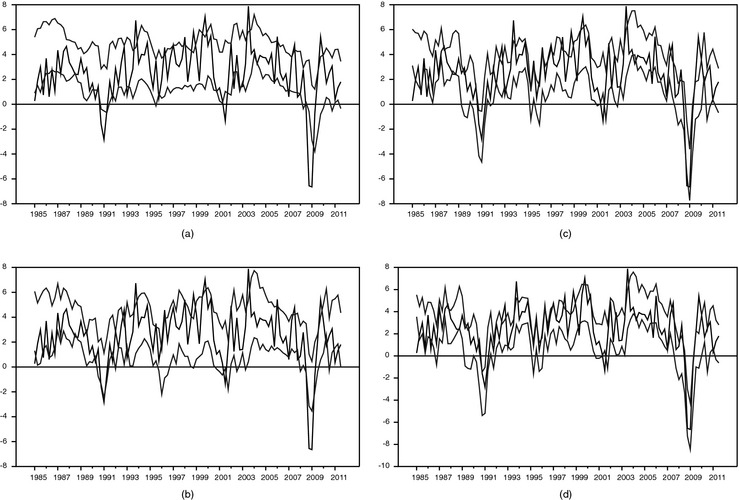
Realtime 70% interval forecasts of GDP growth from the large BMFSV model, 1985, quarter 1–2011, quarter 3 (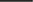, actual GDP; 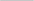, 15th and 85th percentiles): (a) in month 1 of quarter *t*; (b) in month 2 of quarter *t*; (c) in month 3 of quarter *t*; (d) in month 1 of quarter *t*+1

To illustrate the importance of stochastic volatility further, in Figs 3 and 4 we report the realtime 70% interval forecasts from the large BMF model, without (Fig. 3) and with stochastic volatility (Fig. 4). Fig. 3 confirms that the coverage is very poor for models with constant volatilities (estimated recursively). At month 1 of the quarter, for a model with constant volatility, the 70% bands are so wide that actual outcomes hardly ever fall outside the bands. With more months of data, the bands narrow somewhat, but it remains that actual outcomes rarely fall outside the bands. As Fig. 4 indicates, the same model with stochastic volatility yields much narrower bands, and therefore more outcomes that fall outside the 70% bands.

### 5.5 Probability integral transforms

**Figure 5:**
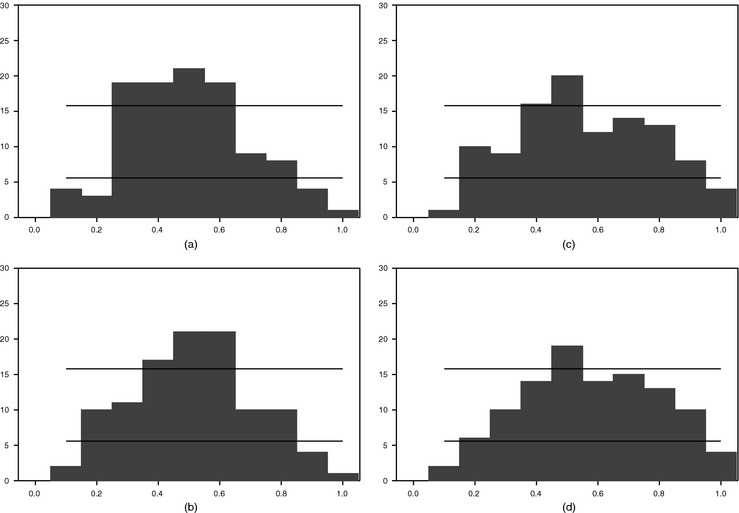
PIT histograms for forecasts of GDP growth for the large BMF model, 1985, quarter 1–2011, quarter 3: (a) in month 1 of quarter *t*; (b) in month 2 of quarter *t*; (c) in month 3 of quarter *t*; (d) in month 1 of quarter *t*+1

**Figure 6:**
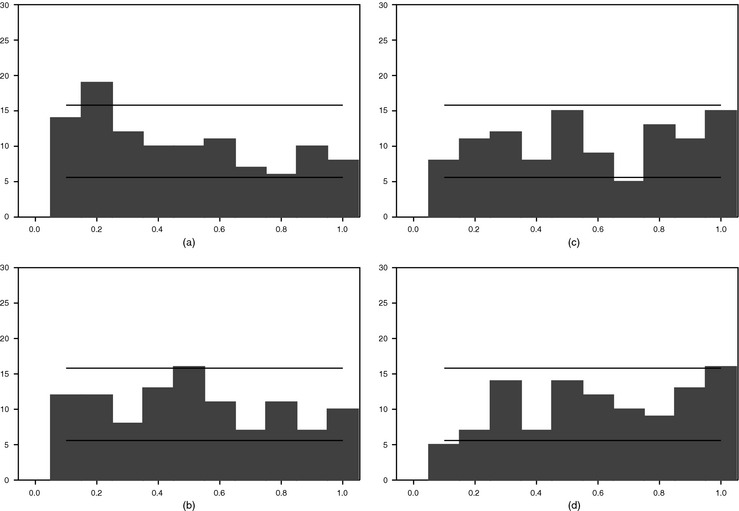
PIT histograms for forecasts of GDP growth for the large BMFSV model, 1985, quarter 1–2011, quarter 3: (a) in month 1 of quarter *t*; (b) in month 2 of quarter *t*; (c) in month 3 of quarter *t*; (d) in month 1 of quarter *t*+1

As noted above, PITs can be seen as a generalization of coverage rates (across different rates). For brevity, we provide in Figs 5 and 6 PIT histograms for just the large BMF and BMFSV models (other models (including AR models) would yield a similar conclusion about the role of stochastic volatility). If the forecasting models were properly specified, the PITs would be uniformly distributed, yielding a completely flat histogram.

The PIT histograms yield results that are in line with the simple coverage comparison of Section 5.4 As Fig. 5 indicates, for models with constant volatilities, the PITs have a distinct tent‐type shape, which is consistent with forecast distributions that are too dispersed. Adding more data does not seem to improve the shape of the PITs materially. This finding provides further evidence that, in the case of models with constant volatilities, the improvement in predictive scores that occurs with the addition of months of data is due to improvement in the forecast mean, not the shape of the distribution.

Fig. 6 shows that including stochastic volatility in the nowcasting model yields much flatter PIT histograms. Hence, by the PITs measure, also, including stochastic volatility materially improves the calibration of density forecasts.

## 6 Conclusions

We have developed a BMF method for producing current quarter forecasts of GDP growth with a (possibly large) range of available within‐the‐quarter monthly observations of economic indicators, such as employment and industrial production, and financial indicators, such as stock prices and interest rates. In light of existing evidence of time variation in the variances of shocks to GDP, we also consider versions of the model with stochastic volatility, whereas most of the existing approaches assumed that the variance is constant.

We use Bayesian methods to estimate the model, to facilitate providing shrinkage on the (possibly large set of) model estimates and conveniently generate predictive densities. Most prior nowcasting research has focused on the accuracy of point forecasts of GDP growth. Instead, we consider both point and density forecasts.

Empirically, we provide results on the accuracy of nowcasts of realtime GDP growth in the USA from 1985 through 2011. In terms of point forecasts, our proposal improves significantly on AR models and performs comparably with survey forecasts, and yields further evidence on the usefulness of intraquarter information. Moreover, our approach provides reliable density and interval forecasts, for which the stochastic volatility specification is quite useful.

Our proposed approach could be extended in several directions, such as using higher frequency information. It could be also applied to nowcast other relevant economic variables, such as components of GDP, the inflation rate or fiscal indicators. We leave these interesting extensions for future research.
